# MOAT-E (ARA) is a full-length MRP/cMOAT subfamily transporter expressed in kidney and liver

**DOI:** 10.1038/sj.bjc.6690527

**Published:** 1999-07

**Authors:** M G Belinsky, G D Kruh

**Affiliations:** Division of Medical Sciences, Fox Chase Cancer Center, 7701 Burholme Avenue, Philadelphia, PA, 19111, USA

**Keywords:** MRP, cMOAT, ABC transporter, resistance

## Abstract

Multidrug resistance-associated protein (MRP) and the canalicular multispecific organic anion transporter (cMOAT) are organic anion pumps that have been linked to cytotoxic drug resistance. We previously reported the isolation of three human MRP/cMOAT-related transporters, MOAT-B (MRP4), MOAT-C (MRP5) and MOAT-D (MRP3). In the present study we describe the fourth MRP/cMOAT-related transporter. We analysed ARA, a human cDNA reported to encode a 453 residue MRP-related transporter, and found that it represents a fused transcript composed of MRP sequences and partial sequences of a novel transporter. The complete coding sequence of this novel transporter, which we designated MOAT-E, was isolated. MOAT-E encodes a 1503 residue transporter that is most closely related to MRP (45%), MOAT-D (44%) and cMOAT (39%), both in terms of amino acid identity and sharing a common topology in which ∼ 17 transmembrane spanning helices are distributed within three membrane spanning domains. RNA blot analysis indicated that MOAT-E expression is restricted to kidney and liver. These observations suggest that MOAT-E may function as an organic anion transporter involved in cellular detoxification and possibly in the hepatobiliary and renal excretion of xenobiotics and/or endogenous metabolites. Isolation of MOAT-E helps to define the MRP/cMOAT subfamily of transporters. © 1999 Cancer Research Campaign


*British Journal of Cancer* (1999) **80**(9), 1342–1349
© 1999 Cancer Research Campaign
Article no. bjoc.1999.0527

### MOAT-E (ARA) is a full-length MRP/cMOAT subfamily transporter expressed in kidney and liver

**MG Belinsky and GD Kruh**

Division of Medical Sciences, Fox Chase Cancer Center, 7701 Burholme Avenue, Philadelphia, PA 19111, USA

**Summary** Multidrug resistance-associated protein (MRP) and the canalicular multispecific organic anion transporter (cMOAT) are organic anion pumps that have been linked to cytotoxic drug resistance. We previously reported the isolation of three human MRP/cMOAT-related transporters, MOAT-B (MRP4), MOAT-C (MRP5) and MOAT-D (MRP3). In the present study we describe the fourth MRP/cMOAT-related transporter. We analysed ARA, a human cDNA reported to encode a 453 residue MRP-related transporter, and found that it represents a fused transcript composed of MRP sequences and partial sequences of a novel transporter. The complete coding sequence of this novel transporter, which we designated MOAT-E, was isolated. MOAT-E encodes a 1503 residue transporter that is most closely related to MRP (45%), MOAT-D (44%) and cMOAT (39%), both in terms of amino acid identity and sharing a common topology in which ~ 17 transmembrane spanning helices are distributed within three membrane spanning domains. RNA blot analysis indicated that MOAT-E expression is restricted to kidney and liver. These observations suggest that MOAT-E may function as an organic anion transporter involved in cellular detoxification and possibly in the hepatobiliary and renal excretion of xenobiotics and/or endogenous metabolites. Isolation of MOAT-E helps to define the MRP/cMOAT subfamily of transporters.

**Keywords:** MRP; cMOAT; ABC transporter; resistance

Cellular resistance mechanisms are a major obstacle to the successful treatment of disseminated malignancies using chemotherapeutic agents. Studies of cell lines made resistant to natural product agents indicate that ATP-binding cassette (ABC) transporters represent important resistance mechanisms associated with these agents. The paradigm for this resistance mechanism is P-glycoprotein (P-gp), which functions as a plasma membrane efflux pump to reduce intracellular levels of natural product cytotoxic agents (Gottesman and Pastan, 1993). More recently, organic anion transporters have been implicated as efflux pumps that confer resistance to natural product cytotoxic agents. The multidrug resistance-associated transporter (MRP), an ABC transporter that shares limited amino acid identity with P-gp (Cole et al, 1992), has been shown to confer a resistance phenotype that overlaps with that of P-gp (Cole et al, 1994; Grant et al, 1994; Kruh et al, 1994; Zaman et al, 1994; Breuninger et al, 1995). In contrast to P-gp, which transports lipophilic amphipathic compounds, MRP functions as an efflux pump for amphipathic anionic conjugates, including glutathione-S conjugates and glucuronidated and sulphated compounds (Leier et al, 1994; Muller et al, 1994; Jedlitschky et al, 1996; Loe et al, 1996). Increasing evidence suggests that an organic anion transporter closely related to MRP, the canalicular multispecific organic acid transporter (cMOAT) (Buchler et al, 1996; Paulusma et al, 1996; Taniguchi et al, 1996), also plays a role in cytotoxic drug resistance. Although the resistance phenotype of cMOAT has not yet been established in transfection studies, cMOAT has been reported to be overexpressed in cell lines selected for resistance to cisplatin (Taniguchi et al, 1996; Kool et al, 1997), and transfection of a cMOAT antisense vector was reported to sensitize a liver cancer cell line to cytotoxic drugs (Koike et al, 1997). In addition, cMOAT-transfected cells have been reported to exhibit enhanced efflux of the natural product drug vincristine (Evers et al, 1998), and a cMOAT-deficient rat strain has been reported to have decreased excretion of methotrexate into the bile (Masuda et al, 1997). Together these studies concerning MRP and cMOAT indicate that organic anion transporters are important in cellular resistance to cytotoxic drugs and possibly the excretion of some of these agents into the bile.

The participation of organic anion transporters in cellular resistance and in the hepatobiliary excretion of cytotoxic drugs suggested that other MRP/cMOAT-related pumps might be relevant to the drug treatment of cancer and analyses in our laboratory (MG Belinsky and GD Kruh, unpublished data) and others (Allikmets et al, 1996; Kool et al, 1997) of expressed sequence tag databases suggested that related human transporters exist. Using a variety of experimental approaches, we previously isolated the full-length coding sequences of three MRP/cMOAT-related transporters, designated MOAT-B, MOAT-C and MOAT-D (Belinsky et al, 1998; Lee et al, 1998). These three transporters correspond to previously reported EST sequences designated MRP4, MRP5 and MRP3 (Allikmets et al, 1996; Kool et al, 1997), respectfully, and in the case of MOAT-C, the partial peptide SMRP (Suzuki et al, 1997). The possibility that an additional MRP/cMOAT subfamily member might exist was suggested by a recent report describing ARA, a cDNA isolated from an anthracycline-resistant cell line and encoding a predicted 453 residue peptide (Longhurst et al, 1996). However, our analysis of the reported ARA cDNA indicated that it represents a fused transcript that encodes a small partial peptide of an MRP/cMOAT-related transporter appended carboxyl-terminal to MRP sequences (MG Belinsky and GD

*Received 30 September 1998*
*Revised 15 January 1999*
*Accepted 19 January 1999*

*Correspondence to:* GD Kruh

**Table 1** Amino acid identity among MRP/cMOAT sub-family members[a]

| | MOAT-E | MOAT-B | MOAT-C<br>percent identity[b] | MOAT-D | MRP | cMOAT |
|---|---|---|---|---|---|---|
| MOAT-E | –<br>– | **33.9**<br>(52.0/56.6) | **30.6**<br>(50.0/52.5) | **43.7**<br>(59.3/59.4) | **45.2**<br>(61.3/61.9) | **38.9**<br>(55.3/59.4) |
| MOAT-B | **33.9**<br>(52.0/56.6) | –<br>– | **36.5**<br>(49.3/59.1) | **35.3**<br>(55.3/54.1) | **39.4**<br>(57.3/61.6) | **36.8**<br>(53.3/55.3) |
| MOAT-C | **30.6**<br>(50.0/52.5) | **36.5**<br>(49.3/59.1) | –<br>– | **33.1**<br>(57.3/56.9) | **35.8**<br>(60.0/59.4) | **36.2**<br>(61.3/60.6) |
| MOAT-D | **43.7**<br>(59.3/59.4) | **35.3**<br>(55.3/54.1) | **33.1**<br>(57.3/56.9) | –<br>– | **57.6**<br>(70.7/73.8) | **46.8**<br>(67.3/70.0) |
| MRP | **45.2**<br>(61.3/61.9) | **39.4**<br>(57.3/61.6) | **35.8**<br>(60.0/59.4) | **57.6**<br>(70.7/73.8) | –<br>– | **48.4**<br>(66.0/73.1) |
| cMOAT | **38.9**<br>(55.3/59.4) | **36.8**<br>(53.3/55.3) | **36.2**<br>(61.3/60.6) | **46.8**<br>(67.3/70.0) | **48.4**<br>(66.0/73.1) | –<br>– |

[a]Overall percent amino acid identity is indicated in bold-face. Percent identity of nucleotide binding folds 1 and 2 is indicated in parentheses (NBF1/NBF2).
[b]Percent identity was obtained using the GAP command in the GCG package.

Kruh, unpublished observations). Since ARA, located at 16p13.1 (Kuss et al, 1998), is in close proximity to MRP (16p13.13), and both of these genes have been reported to be amplified in the drug resistant cell line from which ARA was isolated (Longhurst et al, 1996), it is possible that the ARA cDNA is the product of a rearrangement associated with the MRP amplicon. The detection of cytogenetic abnormalities at 16p in the cell line from which ARA was isolated is consistent with this possibility (O'Neill et al, 1998). Together these observations suggest that ARA encodes a partial peptide of a larger transporter whose full-length cDNA has not yet been described. In the present study we isolate the full-length cDNA of this novel transporter, which we designated MOAT-E. We demonstrate that MOAT-E encodes a 1503 residue ABC transporter that is highly related to MRP, MOAT-D and cMOAT and whose expression is restricted to kidney and liver.

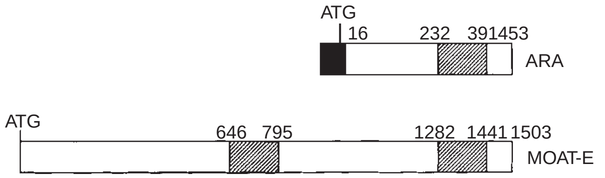

**Figure 1** Schematic comparison of ARA and MOAT-E. The black box indicates MRP nucleotide sequences located at the 5′ end of ARA, and the striped areas denote nucleotide binding folds. The numbering indicates amino acid residues. ARA residues 1–15 are derived from MRP sequence. ARA residues 16–453 (accession number X95715) are nearly identical to MOAT-E carboxyl-terminal residues 999–1503, except as described in the Results section

### MATERIALS AND METHODS

### Isolation of MOAT-E cDNA

Total RNA prepared from human kidney tissue (a gift of Dr D Dexter, Hershey Medical Center) was used to prepare cDNA using the Superscript preamplification system (GibcoBRL, Gaithersberg, MD, USA) according to the manufacturer's instructions. Aliquots of this cDNA were used as template to generate three overlapping PCR fragments, using oligonucleotide primers 5′GGGGCGGCCGCACCATGGCCGCGCCTGCTGAGC3′ (forward) and 5′GTCTACGACACCAGGGTCAAC3′ (reverse); 5′CTGCCTGGAAGAAGTTGACC3′ (forward) and 5′CTGGAATGTCCACGTCAACC3′ (reverse); 5′GGAGACAGACACGGTTGACG3′ (forward) and 5′GCCTCGAGTCAGACCAGGCCTGACTCC3′ (reverse). The first three oligonucleotide primers were designed based upon the predicted exons of a human genomic clone (GenBank accession number U91318), and the last three oligonucleotide primers were based upon the reported sequence of ARA (Longhurst et al, 1996). PCR products were inserted into pBluescript SK- (Stratagene, La Jolla, CA, USA) using the restriction sites located at the 5′ ends of the first and last oligonucleotide primers, and natural restriction sites located within the MOAT-E cDNA. Nucleotide sequence analysis was performed using an ABI 377 DNA sequencer, and the resulting sequences were assembled using the Sequencher program (Gene Codes Corporation, Ann Arbor, MI, USA). Protein sequence analysis was performed using the Wisconsin Package Version 9.1 (Genetics Computer Group, Madison, WI, USA).

### RNA blot analysis

Blots containing poly $A^+$ RNA isolated from human tissues were purchased from Clontech (Palo Alto, CA, USA) and hybridized according to the manufacturer's directions. The 5′ MOAT-E probe encompassed nucleotides 342–656 (amino acids 114–219) and the 3′ MOAT-E probe encompassed nucleotides 3559–4341 (amino acids 1187–1447) of the cDNA.

### RESULTS

### Isolation of MOAT-E cDNA

We analysed the sequence of ARA (Longhurst et al, 1996), a cDNA isolated from a drug-resistant cell line, and found that it is a fused transcript in which 161 nucleotides of MRP sequence are fused to 1775 nucleotides of downstream sequences encoding an MRP/cMOAT-related transporter. The predicted peptide encoded by the downstream sequences was 438 amino acids (residues

*© 1999 Cancer Research Campaign*

**Figure 2** Predicted structure of MOAT-E. Overbars indicate potential transmembrane helices and horizontal arrows indicate the amino-terminal (NBF1) and carboxyl-terminal (NBF2) nucleotide binding folds. The bullet indicates the position of a potential N-glycosylation site conserved with human MRP

16–453), less than one-third the size of other MRP/cMOAT subfamily transporters. A schematic of the predicted fusion protein encoded by the ARA cDNA is shown in Figure 1. These observations suggested that the predicted ARA coding sequence did not represent a protein expressed in normal cells, and that the complete coding sequence of a novel MRP/cMOAT-related transporter remained to be isolated. Consistent with this possibility, database analysis revealed a genomic clone (GenBank accession number

 *© 1999 Cancer Research Campaign*

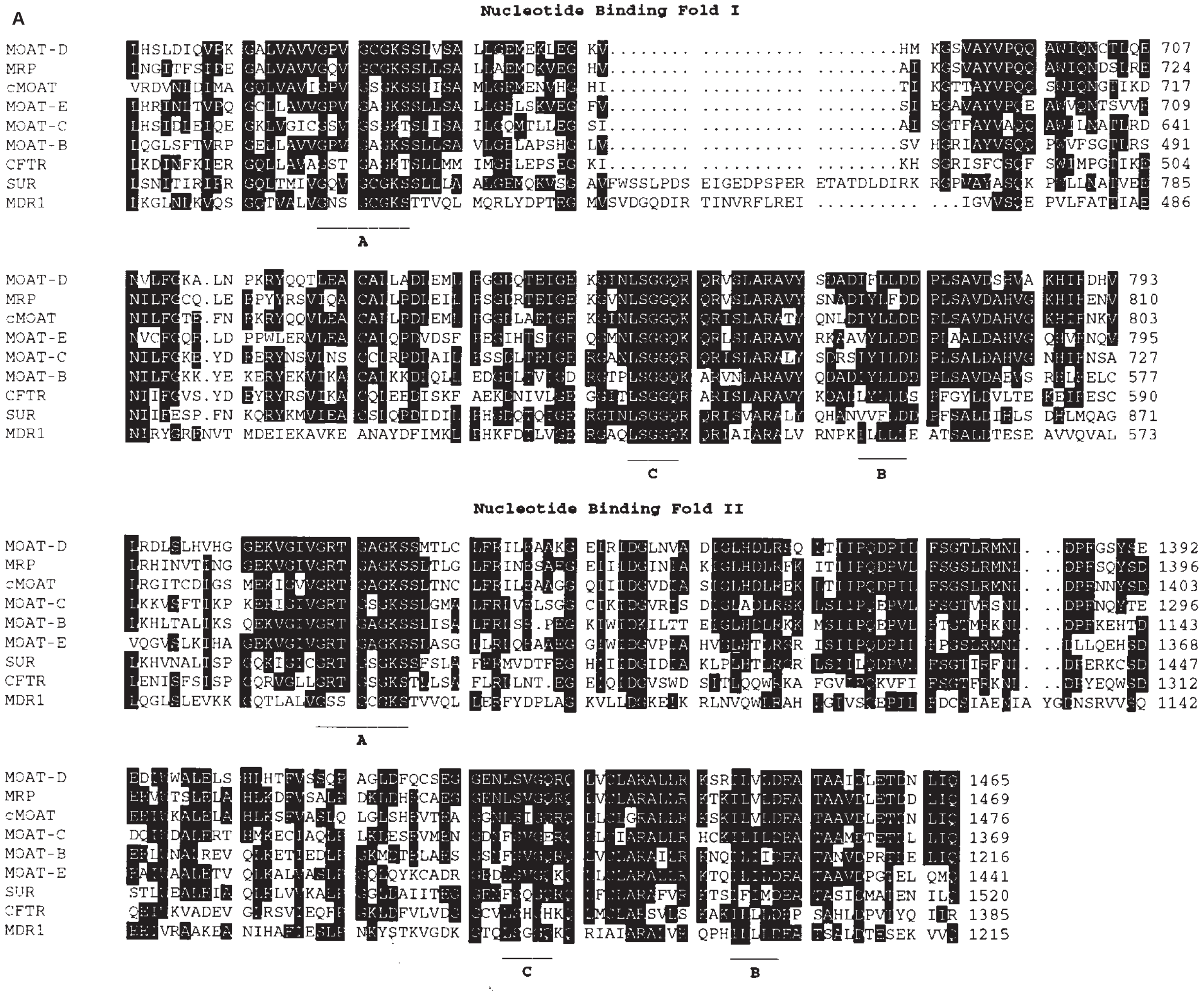

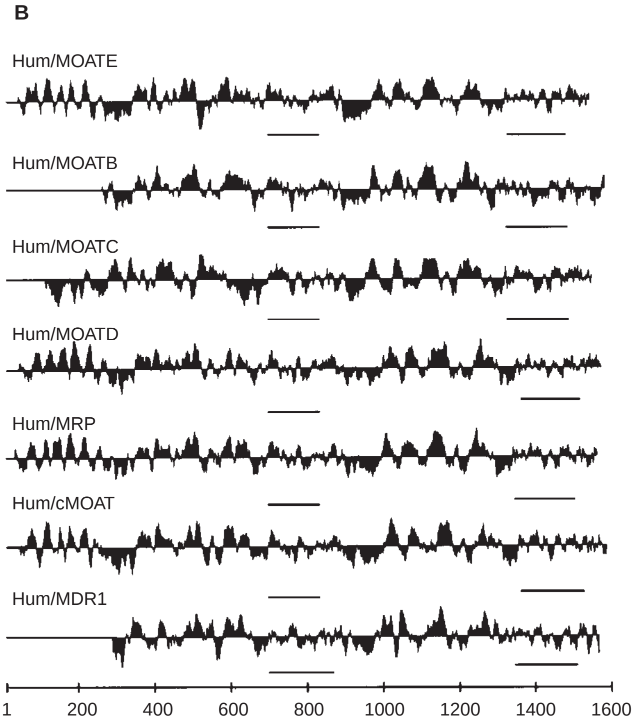

**Figure 3** Comparison of nucleotide binding folds and hydropathy profiles of MOAT-E with those of related ABC transporters. (**A**) Comparison of nucleotide binding folds. The alignment was produced using the PILEUP command (gap weight 3.0, length weight 0.1) in the Genetics Computer Group Package version 9.1. Amino acid positions conserved in at least four of the nine proteins are shaded. In instances where two different amino acids are each conserved in four proteins both of the residues are shaded. Periods indicate gaps in the alignment. Walker A and B motifs, and the ABC transporter family signature sequence C, are indicated by underbars. Accession numbers are indicated in the legend to Figure 5. (**B**) Comparison of hydropathy profiles. Gaps were introduced at the amino-termini of some proteins to bring the amino-terminal nucleotide binding folds into register. Nucleotide binding folds are indicated by horizontal bars. Values above and below the horizontal lines indicate hydrophobic and hydrophilic regions, respectively. Plots were generated using the Kyte–Doolittle algorithm with a window of seven residues

U91318), the predicted exons of which encoded a large potential protein whose carboxyl-terminus was nearly identical to the 438 residue predicted peptide of ARA. To isolate the full-length coding sequence of the peptide encoded by the ARA downstream sequences, we used a reverse transcriptase polymerase chain reaction (RT-PCR) approach in which cDNA prepared from human kidney RNA served as template for oligonucleotide primers that were designed based upon genomic clone U91318 and the reported ARA sequence. This approach yielded a total of ~ 4.5 kb of overlapping cDNA clones. Nucleotide sequence analysis revealed an open reading frame of 1503 residues, the predicted protein of which we designated MOAT-E.

*© 1999 Cancer Research Campaign*

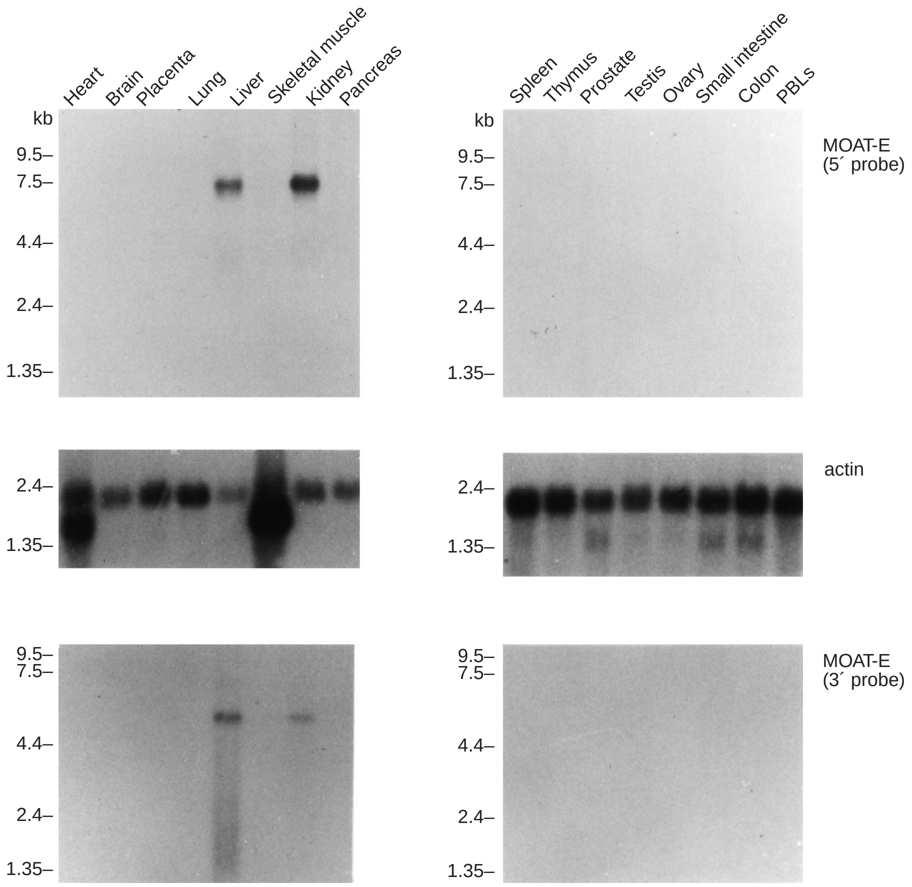

**Figure 4** Tissue distribution of MOAT-E transcript. Membranes containing poly-A+ RNA prepared from various human tissues were hybridized with a probe derived from the 5′ end of the MOAT-E cDNA (top panel). The same membranes were also hybridized with a control actin probe (middle panel). The bottom panel shows a different set of membranes hybridized with a probe derived from 3′ MOAT-E sequences that are also present in ARA

### Analysis of the MOAT-E predicted protein

The predicted amino acid sequence of MOAT-E is shown in Figure 2. Typical features of ABC transporters are present in MOAT-E. Overall, the protein is composed of hydrophobic membrane spanning domains and two nucleotide binding folds (NBFs). Conserved Walker A and B ATP binding motifs, and a conserved C motif, the signature sequence of ABC transporters, are present in the NBFs. As shown schematically in Figure 1, the carboxyl-terminus of the MOAT-E predicted protein (residues 999–1503) corresponds to the MRP-related peptide encoded by ARA (residues 16–453). However, the respective amino acid sequences are not completely identical (96%). Compared to MOAT-E, ARA harbours a small deletion and four amino acid substitutions. MOAT-E residues 1080–1168 are absent in ARA, and MOAT-E residues A-1215, L-1287, L-1335 and S-1386, are represented in ARA by threonine, phenylalanine, valine and cysteine residues respectively.

NBFs are conserved features of ABC transporters, and the degree of similarity between the NBFs of family members indicates the potential for functional conservation (Higgins, 1992). Comparison of the NBFs of MOAT-E with other human ABC transporters indicated that they were most closely related to MRP, cMOAT and three MRP/cMOAT-related transporters we recently described, MOAT-B, MOAT-C and MOAT-D (Table 1) (Belinsky et al, 1998; Lee et al, 1998). Among these transporters, the NBFs of MOAT-E (NBF1/NBF2) shared the highest degree of identity with those of MRP (61/62%), MOAT-D (59/59%) and cMOAT (55/59%). A comparison of the amino acid sequences of the MOAT-E NBFs with those of related transporters is shown in Figure 3A. A distinguishing feature highlighted by these alignments is the presence of small insertions in the NBF1 of SUR and MDR1 that are absent in the NBF1 of MOAT-E, other MRP/cMOAT subfamily members, and CFTR. Consistent with the analysis of NBFs, overall the MOAT-E predicted protein shared the highest degree of amino acid identity with MRP (45%), MOAT-D (44%) and cMOAT (39%) (Table 1). MOAT-E was less well related to MOAT-B and MOAT-C, with which it shared 34% and 31% identity respectively.

A comparison of the hydropathy profiles of MOAT-D with other MRP/cMOAT subfamily transporters, and P-gp, is shown in Figure 3B. Similar to MRP, MOAT-D and cMOAT, MOAT-E has three membrane spanning domains, including an amino-terminal membrane spanning domain that is not present in MOAT-B, MOAT-C or most other ABC transporters, such as P-gp. A 5 + 6 + 6 configuration of transmembrane spanning helices has been proposed for MRP and cMOAT, in which the amino-terminal membrane spanning domain harbours five transmembrane spanning helices, and six transmembrane helices are located in both the second and third membrane spanning domains (Bakos et al, 1996;

© 1999 Cancer Research Campaign

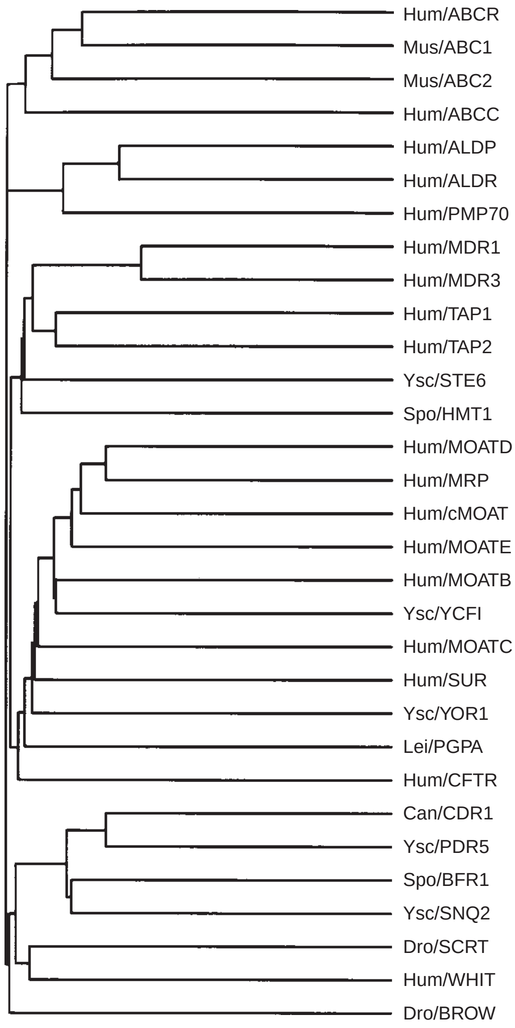

**Figure 5** Cluster analysis of selected eukaryotic ABC transporters. The alignment was generated using the PILEUP command in the GCG package. Accession numbers are as follows: Hum/ABCR: AF000148; Mus/ABC1: X75926; Mus/ABC2: X75927; Hum/ABCC: X97187; Hum/ALDP: P33897; Hum/ALDR: AJ000327; Hum/PMP70: P28288; Hum/MDR1: P08183; Hum/MDR3: M23234; Hum/TAP1: Q03518; Hum/TAP2: 106985; Ysc/STE6: M26376; Spo/HMT1: Q02592; Hum/MRP: P33527; Hum/cMOAT: U63970; Hum/MOATB: AF071202; Ysc/YCF1: P39109; Hum/SUR: Q09428; Ysc/YOR1: P53049; Lei/PGPA: P21441; Hum/CFTR: P13569; Can/CDR1: P43071; Ysc/PDR5: L19922; Spo/BFR1: D55697; Ysc/SNQ2: P32568; Dro/SCRT: U39739; Hum/WHIT: P45844; Dro/BROW: P12428

Hipfner et al, 1997; Kast and Gros, 1997; Tusnady et al, 1997). The structure of MOAT-D is consistent with this (Belinsky et al, 1998). A combination of computer-assisted analysis using the TMAP program (Persson and Argos, 1994) and inspection of an alignment of MOAT-E with MRP, indicated that a 5 + 6 + 6 configuration of transmembrane helices is possible for MOAT-E. This configuration of transmembrane spanning helices is shown in Figure 2. Using computer-assisted analysis alone another configuration of transmembrane spanning helices was predicted (5 + 6 + 4). MRP has been reported to have two N-linked glycosylation sites in its amino-terminus, and another site located between the first and second transmembrane spanning helix of its third membrane spanning domain (Hipfner et al, 1997). A potential amino-terminal N-glycosylation site was conserved in MOAT-E (Asn-15).

### Expression pattern of MOAT-E

ARA was reported to be expressed as a 2.2 kb transcript in the anthracycline resistant cell line from which it was isolated (Longhurst et al, 1996). However, an RNA blot of normal human tissues using ARA sequences as a probe has not been reported. If MOAT-E represents the normal transcript of ARA, we hypothesized that it should be expressed as a larger sized transcript. To test this hypothesis, and gain insight into the possible function of MOAT-E, its expression pattern in a variety of human tissues was examined. Figure 4 (upper panel) shows an RNA blot using a MOAT-E probe derived from 5′ sequences of the cDNA that are not present in ARA. As expected, a MOAT-E transcript (~ 6 kb) that is considerably larger than the reported ARA transcript was detected. In addition, MOAT-E expression was strikingly restricted. Of the 16 tissues analysed, MOAT-E transcript was detected only in liver and kidney. This expression pattern suggests that MOAT-E subserves a specialized function in these two excretory tissues. To confirm that an identical 6 kb transcript was detected using a probe derived from sequences present in ARA, another set of membranes containing RNAs prepared from human tissues was hybridized with a 3′ MOAT-E probe. As shown in Figure 4 (lower panel) this probe also detected 6 kb transcripts in liver and kidney (some degradation is evident in the liver sample).

### MOAT-E helps to define the MRP/cMOAT evolutionary cluster

A cluster analysis of eukaryotic ABC transporters generated using the PILEUP program of the GCG group package is shown in Figure 5. This analysis indicates that the known eukaryotic ABC transporters fall into five families. The MRP/cMOAT subfamily transporters (MRP, cMOAT, MOAT-B, MOAT-C, MOAT-D and MOAT-E) reside within a single cluster, which currently contains the largest number of human ABC transporters. The close relationship between MRP, cMOAT, MOAT-D and MOAT-E is reflected in the close grouping of these four transporters within the cluster. Also within the MRP/cMOAT cluster are the yeast transporters YCF1 and YOR1, and the leishmania transporter PGPA. Like MRP and cMOAT, these three proteins have been reported to transport organic anions (Ouellette et al, 1990; Gui et al, 1996; Li et al, 1996). However, two proteins that are not known to function as organic anion transporters also reside within this cluster. The cystic fibrosis transmembrane conductance regulator, CFTR, functions as an ATP-regulated chloride channel, and the sulphonyl urea receptor, SUR, functions to regulate potassium channels.

### DISCUSSION

We previously reported the complete coding sequences of MOAT-B (MRP4), MOAT-C (MRP5) and MOAT-D (MRP3), three MRP/cMOAT subfamily members (Belinsky et al, 1998; Lee et al, 1998). Based upon the degree of amino acid identity and overall protein topology, we found that the MRP/cMOAT subfamily could be divided into two groups. The first group was composed of MRP, cMOAT and MOAT-D, three transporters that share a high degree of amino acid identity (47–57%), and a common topology characterized by a third membrane spanning domain located at their amino-termini. In contrast, MOAT-B and MOAT-C were somewhat less well-related to MRP (39% and 36% respectively) and did not have amino-terminal hydrophobic extensions. The isolation of MOAT-E

© 1999 Cancer Research Campaign now extends the number of full-length MRP/cMOAT-related transporters to four, and helps to further define this subfamily. Of the known MRP/cMOAT subfamily members, MOAT-E is most closely related to MRP (45%), MOAT-D (44%) and cMOAT (39%). In addition, similar to topological models proposed for the latter three protein, analysis of the primary structure of MOAT-E suggests a model in which 17 transmembrane spanning helices are distributed within three membrane spanning domains in a 5 + 6 + 6 configuration. Thus, MOAT-E belongs to the first group of MRP/cMOAT-transporters we described. Two recently reported transporters isolated from rat liver, MLP-1 and MLP-2 (Hirohashi et al, 1998), are orthologues of MOAT-E (81.5% identity) and MOAT-D (79.6% identity), respectively.

Based upon the close relationship of MOAT-E to MRP and cMOAT, we speculate that it may also function as an organic anion transporter. MRP has been reported to transport a variety of amphiphilic conjugates, including several glutathione S-conjugates, such as the endogenous substrates $LTC_4$ and oxidized glutathione, and xenobiotic conjugates such as DNP-glutathione and monoglutathionyl melphalan (Leier et al, 1994, 1996; Muller et al, 1994). In addition, several sulphated and glucuronidated compounds are MRP substrates (Jedlitschky et al, 1996; Loe et al, 1996). In the case of cMOAT, genetic and biochemical studies of rat strains that are deficient in this protein originally indicated that it functions as an important transporter of amphipathic conjugates into bile (Jansen et al, 1985; Mikami et al, 1986). More recently, several studies using the cloned cMOAT cDNA have confirmed this substrate specificity. Transient expression of rat cMOAT in COS cells and *Xenopus laevis* oocytes has been reported to induce increased cellular efflux of 2,4-dinitrophenyl-S-glutathione and $LTC_4$ (Madon et al, 1997). In addition, enhanced ATP-dependent uptake of glutathione S-conjugates by cMOAT-enriched membrane vesicles prepared from insect cells (Remon et al, 1998) and stably transfected cells (Madon et al, 1997; Evers et al, 1998; Ito et al, 1998) has been reported. Studies using the MOAT-E cDNA should determine whether it shares the substrate specificity of MRP and cMOAT, or possibly transports a distinct class of compounds.

Using RNA blot analysis, we found that MOAT-E transcript was abundant in liver and kidney, but undetectable in many other human tissues. This expression pattern is distinct from those of other MRP/cMOAT subfamily transporters. MRP and MOAT-C are widely expressed, cMOAT is expressed at high levels in the liver, and low levels in small intestine and kidney, MOAT-B is highly expressed in prostate, but also expressed in other tissues, and MOAT-D is expressed in colon, pancreas, liver and kidney, with lower levels in small intestine, prostate and placenta (Kruh et al, 1995; Buchler et al, 1996; Paulusma et al, 1996; Kool et al, 1997; Suzuki et al, 1997; Schaub et al, 1997; Belinsky et al, 1998; Lee et al, 1998). The MOAT-E expression pattern suggests that it may participate in hepatobiliary and renal excretion of organic anions. While cMOAT is a major pump for organic anions in liver, the hepatobiliary excretion of organic anions is not completely abolished in cMOAT-deficient rat strains, suggesting the existence of other organic anion transporters. MRP is expressed in hepatocytes, but its level is low, and it is localized at the lateral membrane which does not communicate with bile canaliculi (Mayer et al, 1995). MOAT-E may therefore function as an alternative system to cMOAT for the hepatobiliary excretion of organic anions. It is also possible, however, that MOAT-E subserves a different function in the liver. The abundant expression of MOAT-E transcript in kidney is particularly interesting. While cMOAT is expressed in the kidney, its expression level is low, and the urinary excretion of organic anions has been reported to be largely unaffected in cMOAT-deficient TR- rats (Huber et al, 1987; de Vries et al, 1989). Thus MOAT-E may function as an ATP-dependent transporter of organic anions into urine. The excretion of organic anions into urine is particularly relevant to methotrexate. A role for cMOAT in the hepatobiliary excretion of this agent has been proposed based upon the observation that cMOAT-deficient mice have increased plasma levels, and decreased biliary excretion of this agent (Masuda et al, 1997). However, in humans methotrexate is predominately excreted into the urine (Allegra and Grem, 1997). MOAT-E is therefore a potential candidate for the renal transporter involved in the urinary excretion of this agent. MOAT-D is also well-expressed in kidney (Belinsky et al, 1998) and may therefore also play a role in organic anion excretion by the kidney. Additional studies should determine whether MOAT-E is involved in the renal and hepatobiliary excretion of organic anions, and whether it plays a role in the cellular detoxification of natural product cytotoxic drugs.

### ACKNOWLEDGEMENTS

This work was supported by NIH grant CA63173 and ACS grant DHP-131 (to G.D.K.), NIH grant CA06927 to Fox Chase Cancer Center and by an appropriation from the Commonwealth of Pennsylvania.

We thank Dr Dwayne Dexter for the gift of human liver and kidney RNA and Avidon Apel for assistance with plasmid preparations and nucleotide sequence analysis.

Two other groups have recently reported the complete MOAT-D/MRP3 coding sequence: Kiuchi et al (1998) *FEBS Lett* **433**: 149–152 and Uchiumi et al (1998) *Biochem Biophys Res Commun* **252**: 103–110. Following submission of our manuscript, the MRP6 coding sequence was reported by Kool et al (1999) *Cancer Res* **59**: 175–182. The MRP6 coding sequence is identical to the MOAT-E sequence with the exception of two amino acid residues.

### REFERENCES

Allegra CJ and Grem JL (1997) Antimetabolites. In: *Cancer, Principles and Practice of Oncology*, DeVita VT Jr, Hellman S and Rosenberg SA (eds), Lippincott-Raven: Philadelphia

Allikmets R, Gerrard B, Hutchinson A and Dean M (1996) Characterization of the human ABC superfamily: isolation and mapping of 21 new genes using the expressed sequence tags database. *Hum Mol Genet* **5**: 1649–1655

Bakos E, Hegedus T, Hollo Z, Welker E, Tusnady GE, Zaman GJ, Flens MJ, Varadi A and Sarkadi B (1996) Membrane topology and glycosylation of the human multidrug resistance-associated protein. *J Biol Chem* **271**: 12322–12326

Belinsky MG, Bain LJ, Balsara BB, Testa JR and Kruh GD (1998) Characterization of MOAT-C and MOAT-D, new members of the MRP/cMOAT subfamily of transporter proteins. *J Natl Cancer Inst* **90**: 1735–1741

Breuninger LM, Paul S, Gaughan K, Miki T, Chan A, Aaronson SA and Kruh GD (1995) Expression of multidrug resistance-associated protein in NIH/3T3 cells confers multidrug resistance associated with increased drug efflux and altered intracellular drug distribution. *Cancer Res* **55**: 5342–5347

Buchler M, Konig J, Brom M, Kartenbeck J, Spring H, Horie T and Keppler D (1996) cDNA cloning of the hepatocyte canalicular isoform of the multidrug resistance protein, cMrp, reveals a novel conjugate export pump deficient in hyperbilirubinemic mutant rats. *J Biol Chem* **271**: 15091–15098

Cole SP, Bhardwaj G, Gerlach JH, Mackie JE, Grant CE, Almquist KC, Stewart AJ, Kurz EU, Duncan AM and Deeley RG (1992) Overexpression of a transporter gene in a multidrug-resistant human lung cancer cell line [see comments]. *Science* **258**: 1650–1654

Cole SP, Sparks KE, Fraser K, Loe DW, Grant CE, Wilson GM and Deeley RG (1994) Pharmacological characterization of multidrug resistant MRP-transfected human tumor cells. *Cancer Res* **54**: 5902–5910

 © 1999 Cancer Research Campaign

Cui Z, Hirata D, Tsuchiya E, Osada H and Miyakawa T (1996) The multidrug resistance-associated protein (MRP) subfamily (Yrs1/Yor1) of *Saccharomyces cerevisiae* is important for the tolerance to a broad range of organic anions. *J Biol Chem* **271**: 14712–14716

de Vries MH, Redegeld FA, Koster AS, Noordhoek J, de Haan JG, Oude Elferink RP and Jansen PL (1989) Hepatic, intestinal and renal transport of 1-naphthol-beta-*D*-glucuronide in mutant rats with hereditary-conjugated hyperbilirubinemia. *Naunyn Schmiedebergs Arch Pharmacol* **340**: 588–592

Evers R, Kool M, van Deemter L, Janssen H, Calafat J, Oomen LC, Paulusma CC, Oude Elferink RP, Baas F, Schinkel AH and Borst P (1998) Drug export activity of the human canalicular multispecific organic anion transporter in polarized kidney MDCK cells expressing cMOAT (MRP2) cDNA. *J Clin Invest* **101**: 1310–1319

Gottesman MM and Pastan I (1993) Biochemistry of multidrug resistance mediated by the multidrug transporter. *Annu Rev Biochem* **62**: 385–427

Grant CE, Valdimarsson G, Hipfner DR, Almquist KC, Cole SP and Deeley RG (1994) Overexpression of multidrug resistance-associated protein (MRP) increases resistance to natural product drugs. *Cancer Res* **54**: 357–361

Higgins CF (1992) ABC transporters: from microorganisms to man. *Annu Rev Cell Biol* **8**: 67–113

Hipfner DR, Almquist KC, Leslie EM, Gerlach JH, Grant CE, Deeley RG and Cole SP (1997) Membrane topology of the multidrug resistance protein (MRP). A study of glycosylation-site mutants reveals an extracytosolic NH2 terminus. *J Biol Chem* **272**: 23623–23630

Huber M, Guhlmann A, Jansen PL and Keppler D (1987) Hereditary defect of hepatobiliary cysteinyl leukotriene elimination in mutant rats with defective hepatic anion excretion. *Hepatology* **7**: 224–228

Ito K, Suzuki H, Hirohashi T, Kume K, Shimizu T and Sugiyama Y (1998) Functional analysis of a canalicular multispecific organic anion transporter cloned from rat liver. *J Biol Chem* **273**: 1684–1688

Jansen PL, Peters WH and Lamers WH (1985) Hereditary chronic conjugated hyperbilirubinemia in mutant rats caused by defective hepatic anion transport. *Hepatology* **5**: 573–579

Jedlitschky G, Leier I, Buchholz U, Barnouin K, Kurz G and Keppler D (1996) Transport of glutathione, glucuronate, and sulfate conjugates by the MRP gene-encoded conjugate export pump. *Cancer Res* **56**: 988–994

Kast C and Gros P (1997) Topology mapping of the amino-terminal half of multidrug resistance-associated protein by epitope insertion and immunofluorescence. *J Biol Chem* **272**: 26479–26487

Koike K, Kawabe T, Tanaka T, Toh S, Uchiumi T, Wada M, Akiyama S, Ono M and Kuwano M (1997) A canalicular multispecific organic anion transporter (cMOAT) antisense cDNA enhances drug sensitivity in human hepatic cancer cells. *Cancer Res* **57**: 5475–5479

Kool M, de Haas M, Scheffer GL, Scheper RJ, van Eijk MJ, Juijn JA, Baas F and Borst P (1997) Analysis of expression of cMOAT (MRP2), MRP3, MRP4, and MRP5, homologues of the multidrug resistance-associated protein gene (MRP1), in human cancer cell lines. *Cancer Res* **57**: 3537–3547

Kruh GD, Chan A, Myers K, Gaughan K, Miki T and Aaronson SA (1994) Expression complementary DNA library transfer establishes mrp as a multidrug resistance gene. *Cancer Res* **54**: 1649–1652

Kruh GD, Gaughan KT, Godwin A and Chan A (1995) Expression pattern of MRP in human tissues and adult solid tumor cell lines. *J Natl Cancer Inst* **87**: 1256–1258

Kuss BJ, O'Neill GM, Eyre H, Doggett NA, Callen DF and Davey RA (1998) ARA, a novel ABC transporter, is located at 16p13.1, is deleted in inv(16) leukemias, and is shown to be expressed in primitive hematopoietic precursors. *Genomics* **51**: 455–458

Lee K, Belinsky MG, Bell DW, Testa JR and Kruh GD (1998) Isolation of MOAT-B, a widely expressed multidrug resistance-associated protein/canalicular multispecific organic anion transporter-related transporter. *Cancer Res* **58**: 2741–2747

Leier I, Jedlitschky G, Buchholz U, Cole SP, Deeley RG and Keppler D (1994) The MRP gene encodes an ATP-dependent export pump for leukotriene C4 and structurally related conjugates. *J Biol Chem* **269**: 27807–27810

Leier I, Jedlitschky G, Buchholz U, Center M, Cole SP, Deeley RG and Keppler D (1996) ATP-dependent glutathione disulphide transport mediated by the MRP gene-encoded conjugate export pump. *Biochem J* **314**: 433–437

Li ZS, Szczypka M, Lu YP, Thiele DJ and Rea PA (1996) The yeast cadmium factor protein (YCF1) is a vacuolar glutathione S-conjugate pump. *J Biol Chem* **271**: 6509–6517

Loe DW, Almquist KC, Cole SP and Deeley RG (1996) ATP-dependent 17 beta-estradiol 17-(beta-D-glucuronide) transport by multidrug resistance protein (MRP). Inhibition by cholestatic steroids. *J Biol Chem* **271**: 9683–9689

Longhurst TJ, O'Neill GM, Harvie RM and Davey RA (1996) The anthracycline resistance-associated (ara) gene, a novel gene associated with multidrug resistance in a human leukaemia cell line. *Br J Cancer* **74**: 1331–1335

Madon J, Eckhardt U, Gerloff T, Stieger B and Meier PJ (1997) Functional expression of the rat liver canalicular isoform of the multidrug resistance-associated protein. *FEBS Lett* **406**: 75–78

Masuda M, I'izuka Y, Yamazaki M, Nishigaki R, Kato Y, Ni'inuma K, Suzuki H and Sugiyama Y (1997) Methotrexate is excreted into the bile by canalicular multispecific organic anion transporter in rats. *Cancer Res* **57**: 3506–3510

Mayer R, Kartenbeck J, Buchler M, Jedlitschky G, Leier I and Keppler D (1995) Expression of the MRP gene-encoded conjugate export pump in liver and its selective absence from the canalicular membrane in transport-deficient mutant hepatocytes. *J Cell Biol* **131**: 137–150

Mikami T, Nozaki T, Tagaya O, Hosokawa S, Nakura T, Mori H and Kondou S (1986) The characters of a new mutant in rats with hyperbilirubinuria syndrome. *Congenital Anom.* **26**: 250–251

Muller M, Meijer C, Zaman GJ, Borst P, Scheper RJ, Mulder NH, de Vries EG and Jansen PL (1994) Overexpression of the gene encoding the multidrug resistance-associated protein results in increased ATP-dependent glutathione S-conjugate transport. *Proc Natl Acad Sci USA* **91**: 13033–13037

Neill GM, Peters GB, Harvie RM, MacKenzie HB, Henness S and Davey RA (1998) Amplification and expression of the ABC transporters ARA and MRP in a series of multidrug-resistant leukaemia cell sublines. *Br J Cancer* **77**: 2076–2080

Ouellette M, Fase-Fowler F and Borst P (1990) The amplified H circle of methotrexate-resistant *Leishmania tarentolae* contains a novel P-glycoprotein gene. *Embo J* **9**: 1027–1033

Paulusma CC, Bosma PJ, Zaman GJ, Bakker CT, Otter M, Scheffer GL, Scheper RJ, Borst P and Oude Elferink RP (1996) Congenital jaundice in rats with a mutation in a multidrug resistance-associated protein gene. *Science* **271**: 1126–1128

Persson B and Argos P (1994) Prediction of transmembrane segments in proteins utilising multiple sequence alignments. *J Mol Biol* **237**: 182–192

Remon AMH, van Aubel RA, van Kuijck MA, Koenderink JB, Deen PM, van Os CH and Russel FG (1998) Adenosine triphosphate-dependent transport of anionic conjugates by the rabbit multidrug resistance-associated protein Mrp2 expressed in insect cells. *Mol Pharmacol* **53**: 1062–1067

Schaub TP, Kartenbeck J, Konig J, Vogel O, Witzgall R, Kriz W and Keppler D (1997) Expression of the conjugate export pump encoded by the mrp2 gene in the apical membrane of kidney proximal tubules. *J Am Soc Nephrol* **8**: 1213–1221

Taniguchi K, Wada M, Kohno K, Nakamura T, Kawabe T, Kawakami M, Kagotani K, Okumura K, Akiyama S and Kuwano M (1996) A human canalicular multispecific organic anion transporter (cMOAT) gene is overexpressed in cisplatin-resistant human cancer cell lines with decreased drug accumulation. *Cancer Res* **56**: 4124–4129

Tusnady GE, Bakos E, Varadi A and Sarkadi B (1997) Membrane topology distinguishes a subfamily of the ATP-binding cassette (ABC) transporters. *FEBS Lett.* **402**: 1–3

Zaman GJ, Flens MJ, van Leusden MR, de Haas M, Mulder HS, Lankelma J, Pinedo HM, Scheper RJ, Baas F, Broxterman HJ and Borst P (1994) The human multidrug resistance-associated protein MRP is a plasma membrane drug-efflux pump. *Proc Natl Acad Sci USA* **91**: 8822–8826

*© 1999 Cancer Research Campaign*

## References

[PDF_00544] Allikmets R., Gerrard B., Hutchinson A., Dean M. (1996). Characterization of the human ABC superfamily: isolation and mapping of 21 new genes using the expressed sequence tags database.. Hum Mol Genet.

[PDF_00547] Bakos E, Hegedüs T., Holló Z., Welker E., Tusnády G. E., Zaman G. J., Flens M. J., Váradi A., Sarkadi B. (1996). Membrane topology and glycosylation of the human multidrug resistance-associated protein.. J Biol Chem.

[PDF_00550] Belinsky M. G., Bain L. J., Balsara B. B., Testa J. R., Kruh G. D. (1998). Characterization of MOAT-C and MOAT-D, new members of the MRP/cMOAT subfamily of transporter proteins.. J Natl Cancer Inst.

[PDF_00553] Breuninger L. M., Paul S., Gaughan K., Miki T., Chan A., Aaronson S. A., Kruh G. D. (1995). Expression of multidrug resistance-associated protein in NIH/3T3 cells confers multidrug resistance associated with increased drug efflux and altered intracellular drug distribution.. Cancer Res.

[PDF_00557] Büchler M., König J., Brom M., Kartenbeck J., Spring H., Horie T., Keppler D. (1996). cDNA cloning of the hepatocyte canalicular isoform of the multidrug resistance protein, cMrp, reveals a novel conjugate export pump deficient in hyperbilirubinemic mutant rats.. J Biol Chem.

[PDF_00561] Cole S. P., Bhardwaj G., Gerlach J. H., Mackie J. E., Grant C. E., Almquist K. C., Stewart A. J., Kurz E. U., Duncan A. M., Deeley R. G. (1992). Overexpression of a transporter gene in a multidrug-resistant human lung cancer cell line.. Science.

[PDF_00565] Cole S. P., Sparks K. E., Fraser K., Loe D. W., Grant C. E., Wilson G. M., Deeley R. G. (1994). Pharmacological characterization of multidrug resistant MRP-transfected human tumor cells.. Cancer Res.

[PDF_00570] Cui Z., Hirata D., Tsuchiya E., Osada H., Miyakawa T. (1996). The multidrug resistance-associated protein (MRP) subfamily (Yrs1/Yor1) of Saccharomyces cerevisiae is important for the tolerance to a broad range of organic anions.. J Biol Chem.

[PDF_00578] Evers R., Kool M., van Deemter L., Janssen H., Calafat J., Oomen L. C., Paulusma C. C., Oude Elferink R. P., Baas F., Schinkel A. H. (1998). Drug export activity of the human canalicular multispecific organic anion transporter in polarized kidney MDCK cells expressing cMOAT (MRP2) cDNA.. J Clin Invest.

[PDF_00583] Gottesman M. M., Pastan I. (1993). Biochemistry of multidrug resistance mediated by the multidrug transporter.. Annu Rev Biochem.

[PDF_00585] Grant C. E., Valdimarsson G., Hipfner D. R., Almquist K. C., Cole S. P., Deeley R. G. (1994). Overexpression of multidrug resistance-associated protein (MRP) increases resistance to natural product drugs.. Cancer Res.

[PDF_00588] Higgins C. F. (1992). ABC transporters: from microorganisms to man.. Annu Rev Cell Biol.

[PDF_00590] Hipfner D. R., Almquist K. C., Leslie E. M., Gerlach J. H., Grant C. E., Deeley R. G., Cole S. P. (1997). Membrane topology of the multidrug resistance protein (MRP). A study of glycosylation-site mutants reveals an extracytosolic NH2 terminus.. J Biol Chem.

[PDF_00594] Huber M., Guhlmann A., Jansen P. L., Keppler D. (1987). Hereditary defect of hepatobiliary cysteinyl leukotriene elimination in mutant rats with defective hepatic anion excretion.. Hepatology.

[PDF_00597] Ito K., Suzuki H., Hirohashi T., Kume K., Shimizu T., Sugiyama Y. (1998). Functional analysis of a canalicular multispecific organic anion transporter cloned from rat liver.. J Biol Chem.

[PDF_00600] Jansen P. L., Peters W. H., Lamers W. H. (1985). Hereditary chronic conjugated hyperbilirubinemia in mutant rats caused by defective hepatic anion transport.. Hepatology.

[PDF_00603] Jedlitschky G., Leier I., Buchholz U., Barnouin K., Kurz G., Keppler D. (1996). Transport of glutathione, glucuronate, and sulfate conjugates by the MRP gene-encoded conjugate export pump.. Cancer Res.

[PDF_00606] Kast C., Gros P. (1997). Topology mapping of the amino-terminal half of multidrug resistance-associated protein by epitope insertion and immunofluorescence.. J Biol Chem.

[PDF_00534] Kiuchi Y., Suzuki H., Hirohashi T., Tyson C. A., Sugiyama Y. (1998). cDNA cloning and inducible expression of human multidrug resistance associated protein 3 (MRP3).. FEBS Lett.

[PDF_00609] Koike K., Kawabe T., Tanaka T., Toh S., Uchiumi T., Wada M., Akiyama S., Ono M., Kuwano M. (1997). A canalicular multispecific organic anion transporter (cMOAT) antisense cDNA enhances drug sensitivity in human hepatic cancer cells.. Cancer Res.

[PDF_00613] Kool M., de Haas M., Scheffer G. L., Scheper R. J., van Eijk M. J., Juijn J. A., Baas F., Borst P. (1997). Analysis of expression of cMOAT (MRP2), MRP3, MRP4, and MRP5, homologues of the multidrug resistance-associated protein gene (MRP1), in human cancer cell lines.. Cancer Res.

[PDF_00537] Kool M., van der Linden M., de Haas M., Baas F., Borst P. (1999). Expression of human MRP6, a homologue of the multidrug resistance protein gene MRP1, in tissues and cancer cells.. Cancer Res.

[PDF_00617] Kruh G. D., Chan A., Myers K., Gaughan K., Miki T., Aaronson S. A. (1994). Expression complementary DNA library transfer establishes mrp as a multidrug resistance gene.. Cancer Res.

[PDF_00620] Kruh G. D., Gaughan K. T., Godwin A., Chan A. (1995). Expression pattern of MRP in human tissues and adult solid tumor cell lines.. J Natl Cancer Inst.

[PDF_00622] Kuss B. J., O'Neill G. M., Eyre H., Doggett N. A., Callen D. F., Davey R. A. (1998). ARA, a novel ABC transporter, is located at 16p13.1, is deleted in inv(16) leukemias, and is shown to be expressed in primitive hematopoietic precursors.. Genomics.

[PDF_00626] Lee K., Belinsky M. G., Bell D. W., Testa J. R., Kruh G. D. (1998). Isolation of MOAT-B, a widely expressed multidrug resistance-associated protein/canalicular multispecific organic anion transporter-related transporter.. Cancer Res.

[PDF_00633] Leier I., Jedlitschky G., Buchholz U., Center M., Cole S. P., Deeley R. G., Keppler D. (1996). ATP-dependent glutathione disulphide transport mediated by the MRP gene-encoded conjugate export pump.. Biochem J.

[PDF_00630] Leier I., Jedlitschky G., Buchholz U., Cole S. P., Deeley R. G., Keppler D. (1994). The MRP gene encodes an ATP-dependent export pump for leukotriene C4 and structurally related conjugates.. J Biol Chem.

[PDF_00636] Li Z. S., Szczypka M., Lu Y. P., Thiele D. J., Rea P. A. (1996). The yeast cadmium factor protein (YCF1) is a vacuolar glutathione S-conjugate pump.. J Biol Chem.

[PDF_00639] Loe D. W., Almquist K. C., Cole S. P., Deeley R. G. (1996). ATP-dependent 17 beta-estradiol 17-(beta-D-glucuronide) transport by multidrug resistance protein (MRP). Inhibition by cholestatic steroids.. J Biol Chem.

[PDF_00643] Longhurst T. J., O'Neill G. M., Harvie R. M., Davey R. A. (1996). The anthracycline resistance-associated (ara) gene, a novel gene associated with multidrug resistance in a human leukaemia cell line.. Br J Cancer.

[PDF_00646] Madon J., Eckhardt U., Gerloff T., Stieger B., Meier P. J. (1997). Functional expression of the rat liver canalicular isoform of the multidrug resistance-associated protein.. FEBS Lett.

[PDF_00650] Masuda M., I'izuka Y., Yamazaki M., Nishigaki R., Kato Y., Ni'inuma K., Suzuki H., Sugiyama Y. (1997). Methotrexate is excreted into the bile by canalicular multispecific organic anion transporter in rats.. Cancer Res.

[PDF_00652] Mayer R., Kartenbeck J., Büchler M., Jedlitschky G., Leier I., Keppler D. (1995). Expression of the MRP gene-encoded conjugate export pump in liver and its selective absence from the canalicular membrane in transport-deficient mutant hepatocytes.. J Cell Biol.

[PDF_00659] Müller M., Meijer C., Zaman G. J., Borst P., Scheper R. J., Mulder N. H., de Vries E. G., Jansen P. L. (1994). Overexpression of the gene encoding the multidrug resistance-associated protein results in increased ATP-dependent glutathione S-conjugate transport.. Proc Natl Acad Sci U S A.

[PDF_00663] O'Neill G. M., Peters G. B., Harvie R. M., MacKenzie H. B., Henness S., Davey R. A. (1998). Amplification and expression of the ABC transporters ARA and MRP in a series of multidrug-resistant leukaemia cell sublines.. Br J Cancer.

[PDF_00667] Ouellette M., Fase-Fowler F., Borst P. (1990). The amplified H circle of methotrexate-resistant leishmania tarentolae contains a novel P-glycoprotein gene.. EMBO J.

[PDF_00670] Paulusma C. C., Bosma P. J., Zaman G. J., Bakker C. T., Otter M., Scheffer G. L., Scheper R. J., Borst P., Oude Elferink R. P. (1996). Congenital jaundice in rats with a mutation in a multidrug resistance-associated protein gene.. Science.

[PDF_00674] Persson B., Argos P. (1994). Prediction of transmembrane segments in proteins utilising multiple sequence alignments.. J Mol Biol.

[PDF_00680] Schaub T. P., Kartenbeck J., König J., Vogel O., Witzgall R., Kriz W., Keppler D. (1997). Expression of the conjugate export pump encoded by the mrp2 gene in the apical membrane of kidney proximal tubules.. J Am Soc Nephrol.

[PDF_00684] Taniguchi K., Wada M., Kohno K., Nakamura T., Kawabe T., Kawakami M., Kagotani K., Okumura K., Akiyama S., Kuwano M. (1996). A human canalicular multispecific organic anion transporter (cMOAT) gene is overexpressed in cisplatin-resistant human cancer cell lines with decreased drug accumulation.. Cancer Res.

[PDF_00689] Tusnády G. E., Bakos E., Váradi A., Sarkadi B. (1997). Membrane topology distinguishes a subfamily of the ATP-binding cassette (ABC) transporters.. FEBS Lett.

[PDF_00535] Uchiumi T., Hinoshita E., Haga S., Nakamura T., Tanaka T., Toh S., Furukawa M., Kawabe T., Wada M., Kagotani K. (1998). Isolation of a novel human canalicular multispecific organic anion transporter, cMOAT2/MRP3, and its expression in cisplatin-resistant cancer cells with decreased ATP-dependent drug transport.. Biochem Biophys Res Commun.

[PDF_00692] Zaman G. J., Flens M. J., van Leusden M. R., de Haas M., Mülder H. S., Lankelma J., Pinedo H. M., Scheper R. J., Baas F., Broxterman H. J. (1994). The human multidrug resistance-associated protein MRP is a plasma membrane drug-efflux pump.. Proc Natl Acad Sci U S A.

[PDF_00574] de Vries M. H., Redegeld F. A., Koster A. S., Noordhoek J., de Haan J. G., Oude Elferink R. P., Jansen P. L. (1989). Hepatic, intestinal and renal transport of 1-naphthol-beta-D-glucuronide in mutant rats with hereditary-conjugated hyperbilirubinemia.. Naunyn Schmiedebergs Arch Pharmacol.

[PDF_00676] van Aubel R. A., van Kuijck M. A., Koenderink J. B., Deen P. M., van Os C. H., Russel F. G. (1998). Adenosine triphosphate-dependent transport of anionic conjugates by the rabbit multidrug resistance-associated protein Mrp2 expressed in insect cells.. Mol Pharmacol.

